# Supplementary material for: Genome-Wide Identification and Characterization of the Phytochrome Gene Family in Peanut
Source: Genes (Basel). 2023 Jul 20;14(7):1478. doi: 10.3390/genes14071478 (PMC10378891; doi:10.3390/genes14071478)
Supplement: Supplementary file 1 [file genes-14-01478-s001.zip › Supplementary Materials.pdf]

Supplementary Materials

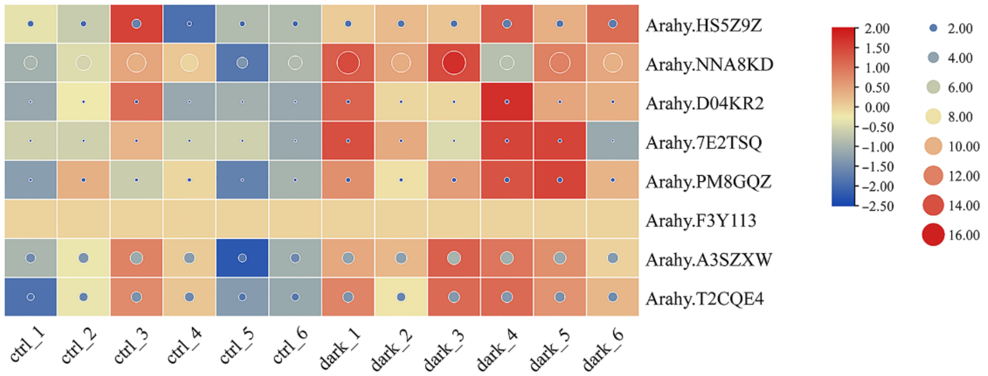

Supplementary Figure S1. Expression profiling of *PHY* genes in cultivated peanut under dark treatment. Aerial grown gynophores ranging from 3 to 5cm in length were subject to complete photo-deprivation for duration of 7 days. The reference genome utilized for mapping the dark-treated transcriptome (unpublished data) was derived from the cultivated peanut in Peanutbase. The square color scale of heatmap indicates TPM values following row normalization, with maximal and minimal expression denoted by red and blue, respectively. The circular color scale indicates raw TPM values, where the area subtended by each circle is directly proportional to the expression level.

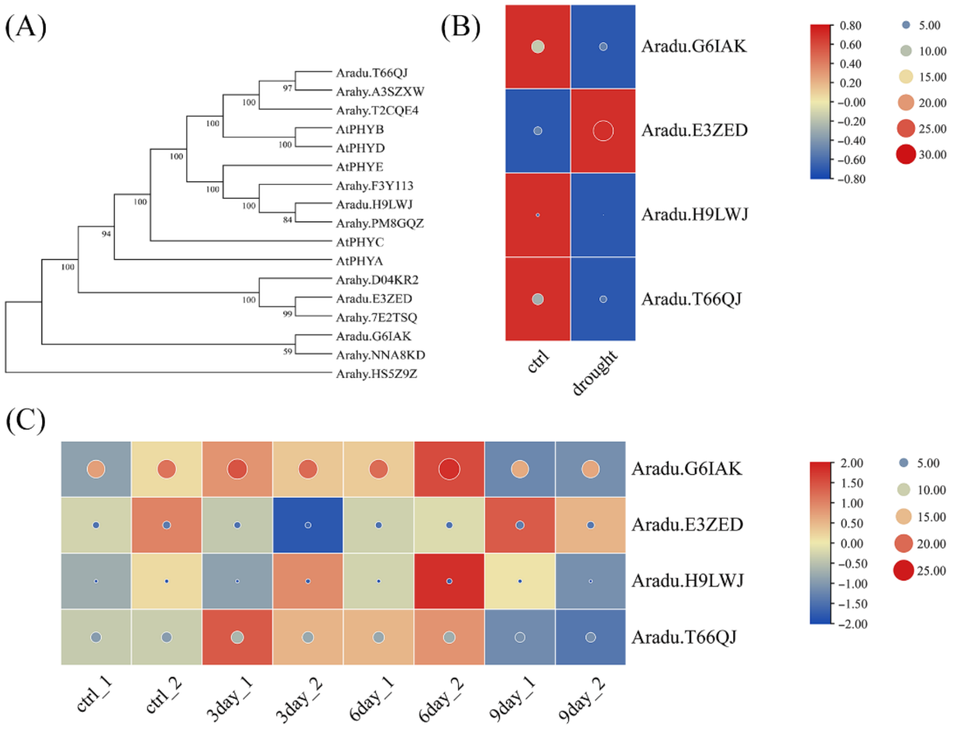

Supplementary Figure S2. Expression profiling of *PHY* genes in wild peanut under abiotic stress. **(A)** Phylogenetic analysis of *PHY* genes from *Arachis duranensis* (AA) *Arachis hypogaea* (AABB) and *Arabidopsis thaliana*. **(B)** Expression profiling of *PHY* genes under drought treatment. **(C)** Expression profiling of *PHY* genes under nematode infection. Transcriptome data for *Arachis duranensis* under drought and nematode treatment were obtained from Peanutbase. The square color scale of heatmap indicates FPKM values following row normalization, with maximal and minimal expression denoted by red and blue, respectively. The circular color scale indicates raw FPKM values, where the area subtended by each circle is directly proportional to the expression level.

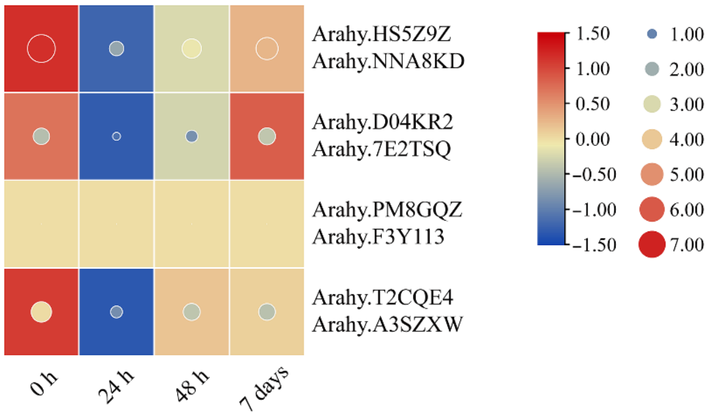

Supplementary Figure S3. Expression profiling of *PHY* genes in cultivated peanut under drought treatment. Young peanut seedlings were treated with 15% PEG6000 solution for 24h, 48h and 7days. The square color scale of heatmap indicates RPKM values following row normalization, with maximal and minimal expression denoted by red and blue, respectively. The circular color scale indicates raw RPKM values, where the area subtended by each circle is directly proportional to the expression level.
